# Supplementary material for: Liver governs adipose remodelling via extracellular vesicles in response to lipid overload
Source: Nat Commun. 2020 Feb 5;11:719. doi: 10.1038/s41467-020-14450-6 (PMC7002740; doi:10.1038/s41467-020-14450-6)
Supplement: Supplementary file 1 — Supplementary Information [file 41467_2020_14450_MOESM1_ESM.pdf]

Supplementary Information

Title: Liver Governs Adipose Remodelling via Extracellular Vesicles in Response to Lipid Overload

Author: Zhao et al

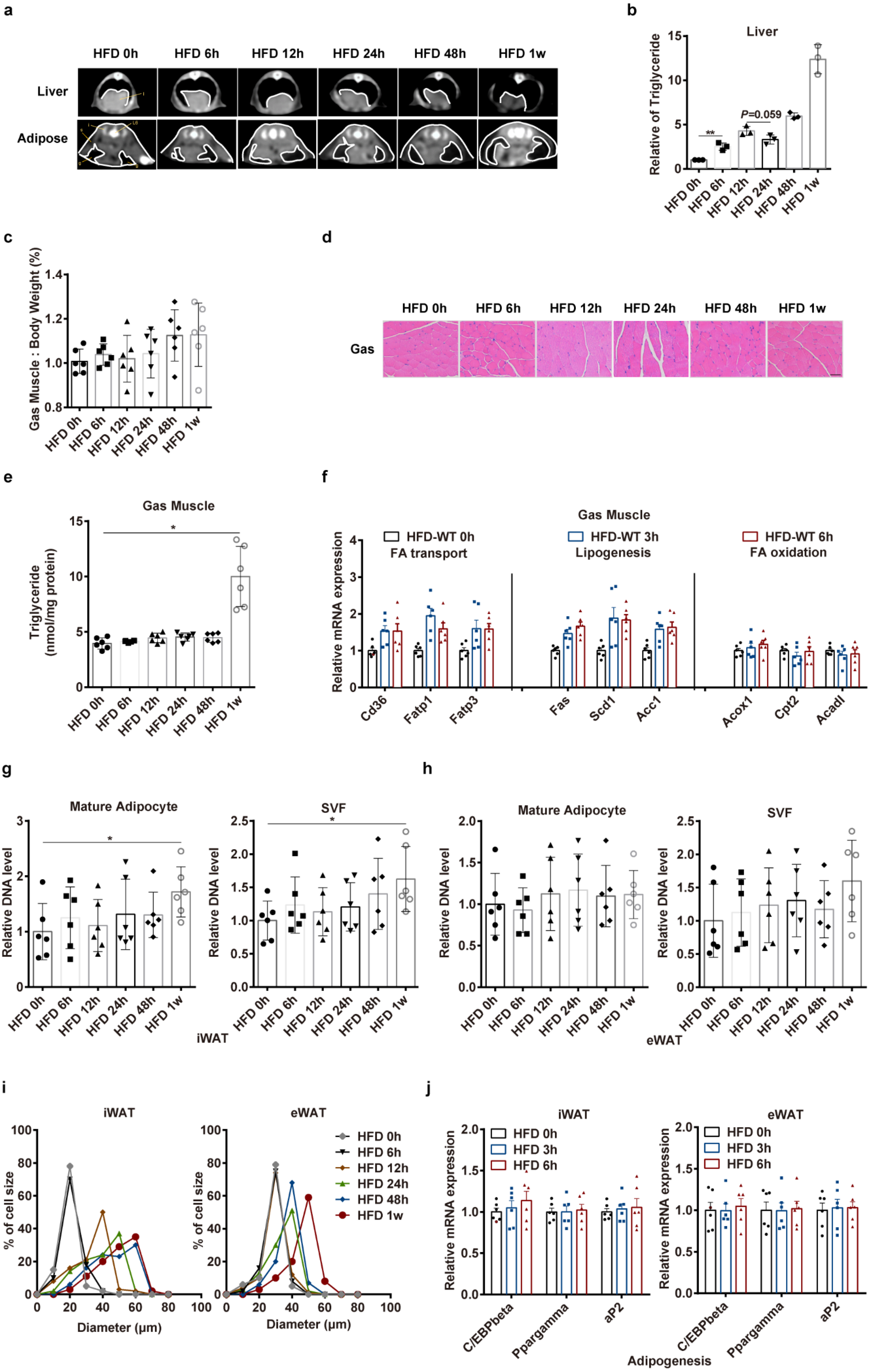

a
HFD 0h
HFD 6h
HFD 12h
HFD 24h
HFD 48h
HFD 1w
Liver
Adipose
b
Liver
Relative of Triglyceride
**
P=0.059
c
Gas Muscle : Body Weight (%)
d
Gas
e
Gas Muscle
Triglyceride (nmol/mg protein)
*
f
Gas Muscle
HFD-WT 0h
HFD-WT 3h
HFD-WT 6h
FA transport
Lipogenesis
FA oxidation
Relative mRNA expression
Cd36
Fatp1
Fatp3
Fas
Scd1
Acc1
Acox1
Cpt2
Acadl
g
Mature Adipocyte
SVF
Relative DNA level
iWAT
h
eWAT
i
iWAT
eWAT
% of cell size
Diameter (μm)
HFD 1w
j
HFD 0h
HFD 3h
HFD 6h
C/EBPbeta
Ppargamma
aP2
Adipogenesis

Supplementary Figure 1. Response of the Liver and Gas Muscle to Lipid Overload. (Related to Fig. 1.)

(**a**) Mice were fed as described in Fig. 1. Representative CT transverse section images are shown. Liver, gonadal fat depots, subcutaneous fat depots, ilia and the 6th lumbar vertebra are indicated as l, g, s, i and L6, respectively. (n = 3 mice per group).

(**b**) The relative TG content in the liver in Supplementary Figure 1a is shown.

(**c**) Percentage of Gas muscle weight relative to the whole-body weight of HFD-fed mice at the indicated time points. (n = 6 mice per group).

(**d**) H&E staining of Gas muscle from HFD-fed mice at the indicated time points (scale bar: 50 μm).

(**e**) TG content in the Gas muscle. (n = 6 mice per group).

(**f**) Expression of genes related to fatty acid transport, lipogenesis and fatty acid oxidation in the Gas muscle of HFD-fed mice at the indicated times. (n = 6 mice per group).

(g-h) Quantification of the number of mature adipocytes and the stromal vascular fraction (SVF) in iWAT and eWAT from HFD-fed mice at the indicated time points. The data reflect the relative total DNA content per fat pad. (n = 6 mice per group).

(**i**) Quantification of the diameter of adipocytes in iWAT and eWAT from HFD-fed mice at the indicated time points. (n = 6 mice per group).

(**j**)   Expression of genes related to adipogensis in the WATs of HFD-fed mice at the indicated times. (n = 6 mice per group).

Data are expressed as the mean ± SEM. **P* < 0.05, ***P* < 0.01, unpaired t test.

Source data are provided as a Source Data file.

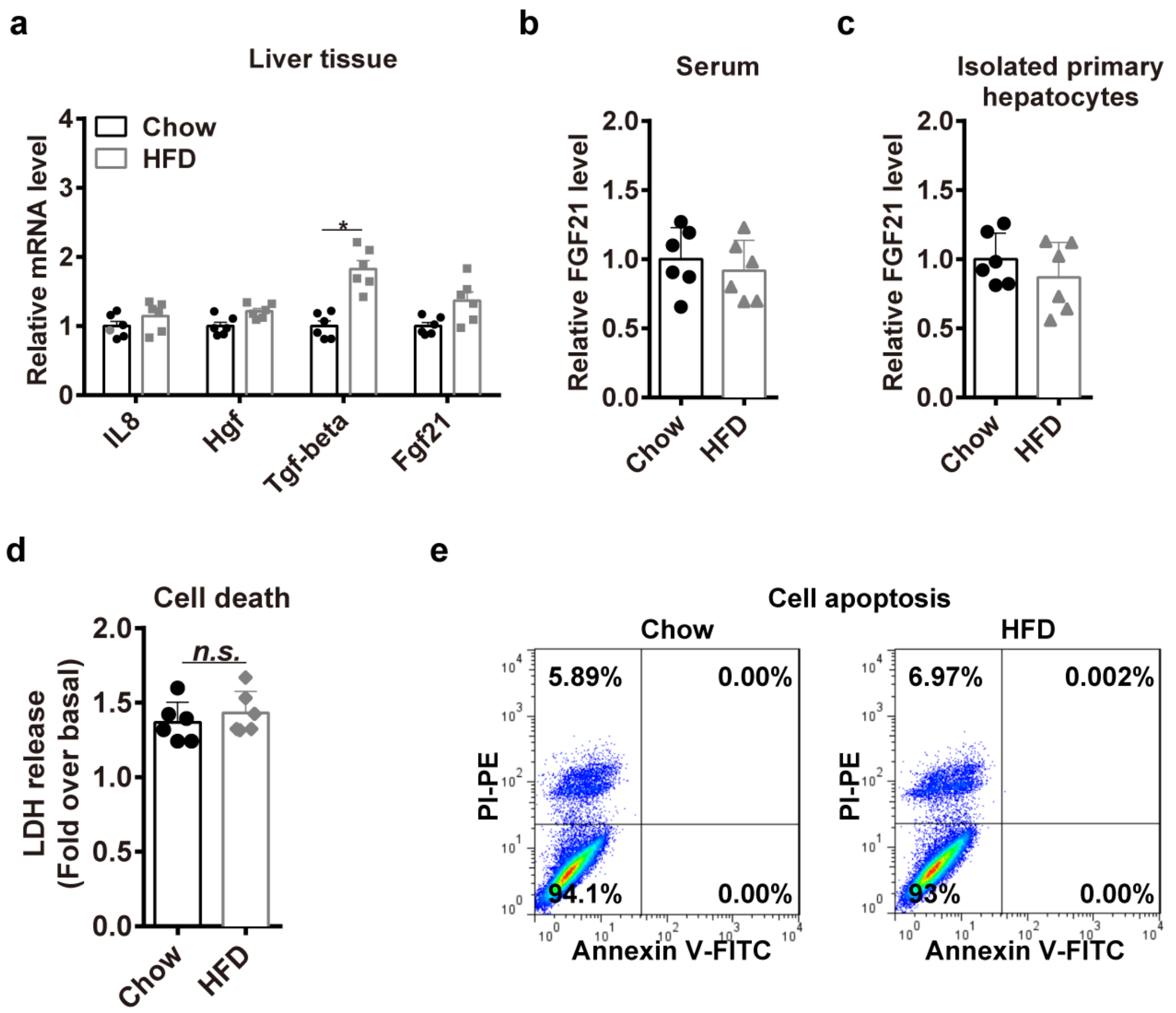

Supplementary Figure 2. Hepatic FGF21 Production and Cell Apoptosis After Lipid Overload. (Related to Fig. 2.)

(**a**) Relative mRNA expression of circulating liver-derived hepatokines and cytokines.

(**b**) Serum FGF21 levels.

(**c**) *Fgf21* mRNA expression in primary hepatocytes isolated from mice fed a normal chow diet or HFD.

(**d**) LDH release by primary hepatocytes isolated from mice fed a normal chow diet or HFD for 6 hours.

(**e**) Apoptosis of primary hepatocytes isolated from mice fed a normal chow diet or HFD for 6 hours.

Data are presented as the mean ± SEM. n = 6 mice per group. *$P < 0.05$, unpaired t test.

Source data are provided as a Source Data file.

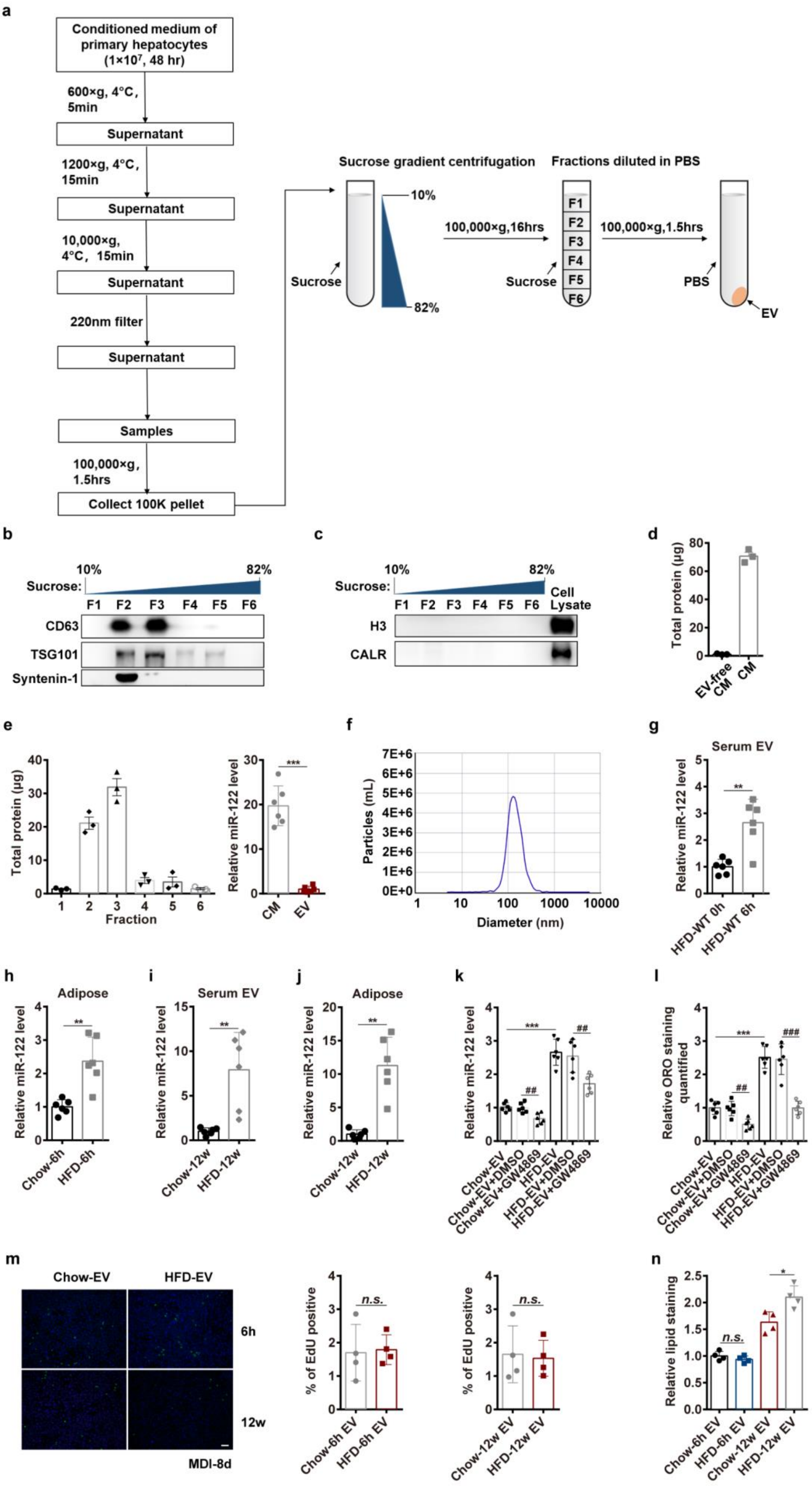
a
Conditioned medium of primary hepatocytes (1×10^7, 48 hr)
600×g, 4°C, 5min
Supernatant
1200×g, 4°C, 15min
Supernatant
10,000×g, 4°C, 15min
Supernatant
220nm filter
Supernatant
Samples
100,000×g, 1.5hrs
Collect 100K pellet
Sucrose gradient centrifugation
10%
82%
Sucrose
100,000×g,16hrs
Fractions diluted in PBS
F1
F2
F3
F4
F5
F6
100,000×g,1.5hrs
PBS
EV
b
Sucrose:
F1 F2 F3 F4 F5 F6
CD63
TSG101
Syntenin-1
c
Cell Lysate
H3
CALR
d
Total protein (μg)
EV-free CM
CM
e
Total protein (μg)
Fraction
Relative miR-122 level
CM
EV
f
Particles (mL)
Diameter (nm)
g
Serum EV
HFD-WT 0h
HFD-WT 6h
h
Adipose
Chow-6h
HFD-6h
i
Serum EV
Chow-12w
HFD-12w
j
Adipose
k
Chow-EV
Chow-EV+DMSO
Chow-EV+GW4869
HFD-EV
HFD-EV+DMSO
HFD-EV+GW4869
l
Relative ORO staining quantified
m
Chow-EV
HFD-EV
6h
12w
MDI-8d
% of EdU positive
n.s.
Chow-6h EV
HFD-6h EV
Chow-12w EV
HFD-12w EV
n
Relative lipid staining

Supplementary Figure 3. Characteristics of Hepatic EVs in Mice After Lipid Overload. (**Related to Fig. 2.)**

(**a**) Schematic of hepatocyte-derived EV isolation.

(**b-c**) Western blot of proteins separated by a sucrose gradient targeting EV markers (**b**) or non-EV proteins (**c**). Each well was loaded with 10 μg of protein.

(**d**) EV abundance in EV-free medium and conditioned medium from the primary hepatocytes described in Supplementary Figure 3a. (n = 3 biologically independent samples per group).

(**e**) Left: EV abundance in subfractions after sucrose gradient flotation (n = 3 biologically independent samples per group); Right: miR-122 levels in CM before differential centrifugation and EV after density gradient centrifugation (n = 6 biologically independent samples per group).

(**f**) Particle characteristics of the vesicles secreted from primary hepatocytes of mice fed a normal chow diet or HFD were measured by using nanoparticle tracking analysis with a Particle Metrix ZetaView®.

(**g**) miR-122 levels in serum EVs from mouse fed an HFD for 6 hours. (n = 6 mice per group).

(**h**) miR-122 levels in adipose tissue from mice fed an HFD for 6 hours. (n = 6 mice per group).

(**i**) miR-122 levels in serum EVs from mice fed a normal chow diet or HFD for 12 weeks (starting at 8 weeks of age). (n = 6 mice per group).

(**j**) miR-122 levels in adipose tissue of mice after 12 weeks of HFD consumption. (n = 6 mice per group).

(**k**) miR-122 levels in 3T3-L1 preadipocytes from Fig. 2f. (n = 6 biologically independent samples per group).

(**l**) Quantification of ORO-positive areas in 3T3-L1 preadipocytes from Fig. 2f. (n = 6 biologically independent samples per group).

(**m**) Quantification of EdU-positive areas in 3T3-L1 preadipocytes from Fig. 2i. (scale bar: 100 μm) (n = 4 biologically independent samples per group).

(**n**) Quantification of lipid content in 3T3-L1 preadipocytes from Fig. 2i. (n = 4 biologically independent samples per group).

Data are presented as the mean ± SEM. Groups were analysed using an unpaired t test, **P* < 0.05, ***P* < 0.01, ****P* < 0.001; ##*P* < 0.01, ###*P* < 0.001.

Source data are provided as a Source Data file.

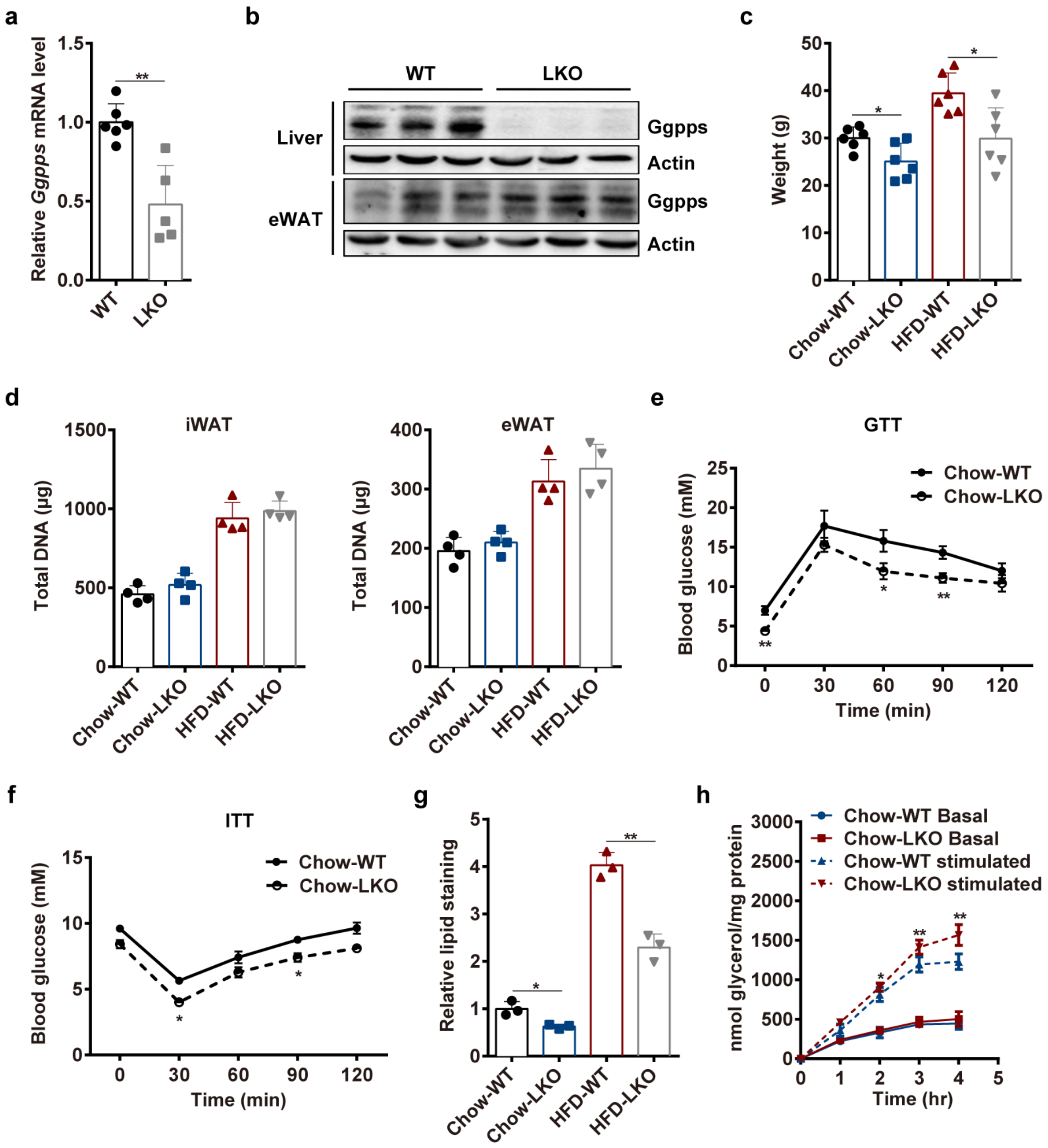

Supplementary Figure 4. *Ggpps* Knockout Efficiency and Characteristics of Adipose Tissue and Metabolism in LKO Mice. (Related to Fig. 3.)

(**a-b**) Real-time PCR (**a**) and Western blot (**b**) analysis of Ggpps expression in livers from WT and LKO mice. (n=6 mice per group).

(**c**) Body weight of the mice in Fig. 3f. (n=6 mice per group).

(**d**) Total amount of DNA in iWAT and eWAT from the WT and LKO mice in Fig. 3f. (n=4 mice per group).

(**e-f**) The glucose tolerance test (GTT) (**e**) and insulin tolerance test (ITT) (**f**) were performed in WT and LKO mice. (n=6 mice per group).

(**g**) Quantification of lipid content in mature adipocytes isolated from iWAT from the mice in Fig. 3f. (n=3 mice per group).

(**h**) Quantification of glycerol from adipocytes isolated from iWAT from the mice in Fig. 3f after induction of lipolysis using isoproterenol. (n=3 mice per group).

Data are presented as the mean ± SEM. **P* < 0.05, ***P* < 0.01, unpaired t test.

Source data are provided as a Source Data file.

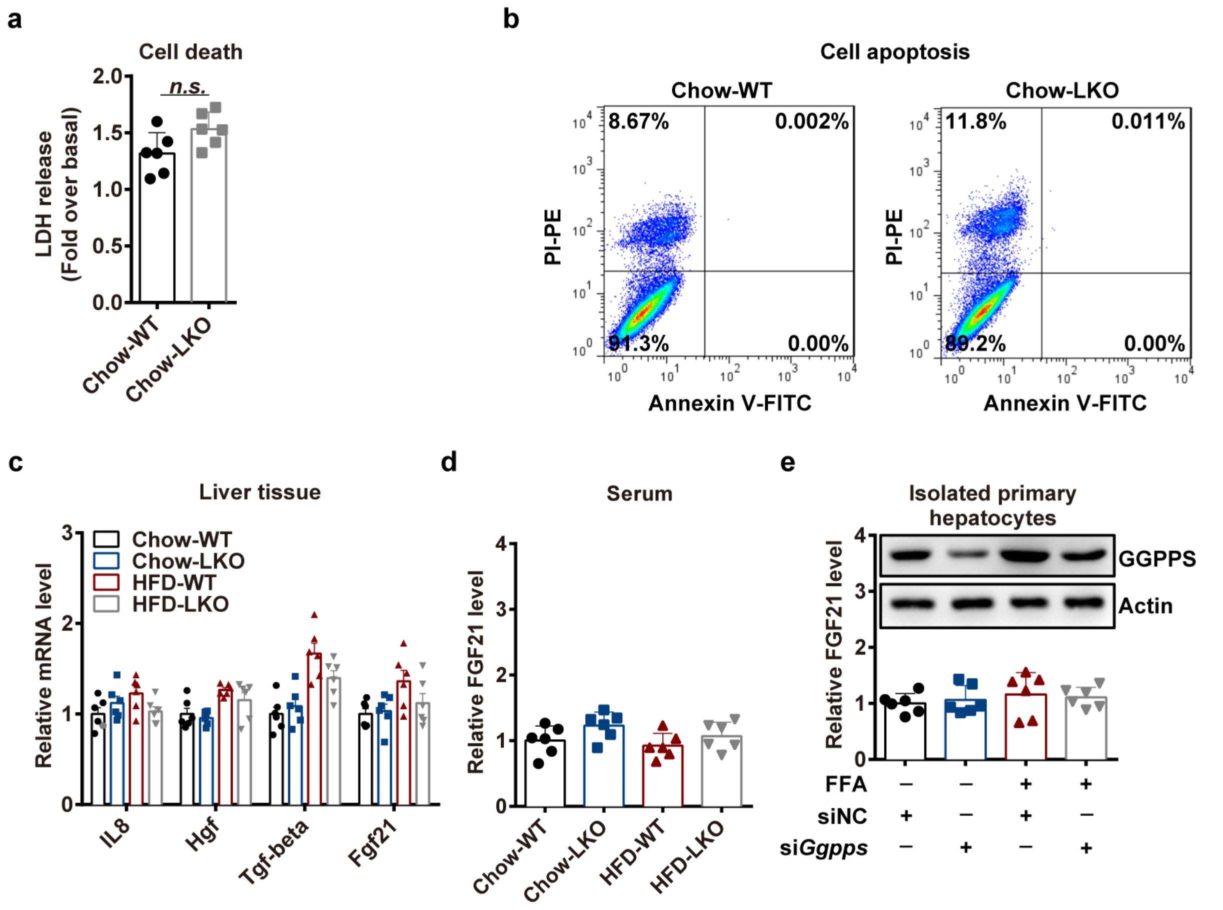

Supplementary Figure 5. Cell Viability and Hepatokine Production in LKO mice. (Related to Fig. 4.)

(**a**) LDH release by primary hepatocytes isolated from WT and LKO mice.

(**b**) Apoptosis of primary hepatocytes isolated from WT and LKO mice.

(**c**) Relative mRNA expression of circulating liver-derived hepatokines and cytokines.

(**d**) Serum FGF21 levels.

(**e**) Ggpps protein expression and *Fgf21* mRNA expression in primary hepatocytes treated with si*Ggpps* in the presence or absence of FFA treatment.

Data are presented as the mean ± SEM. n = 6 mice per group. Groups were analysed using an unpaired t test.

Source data are provided as a Source Data file.

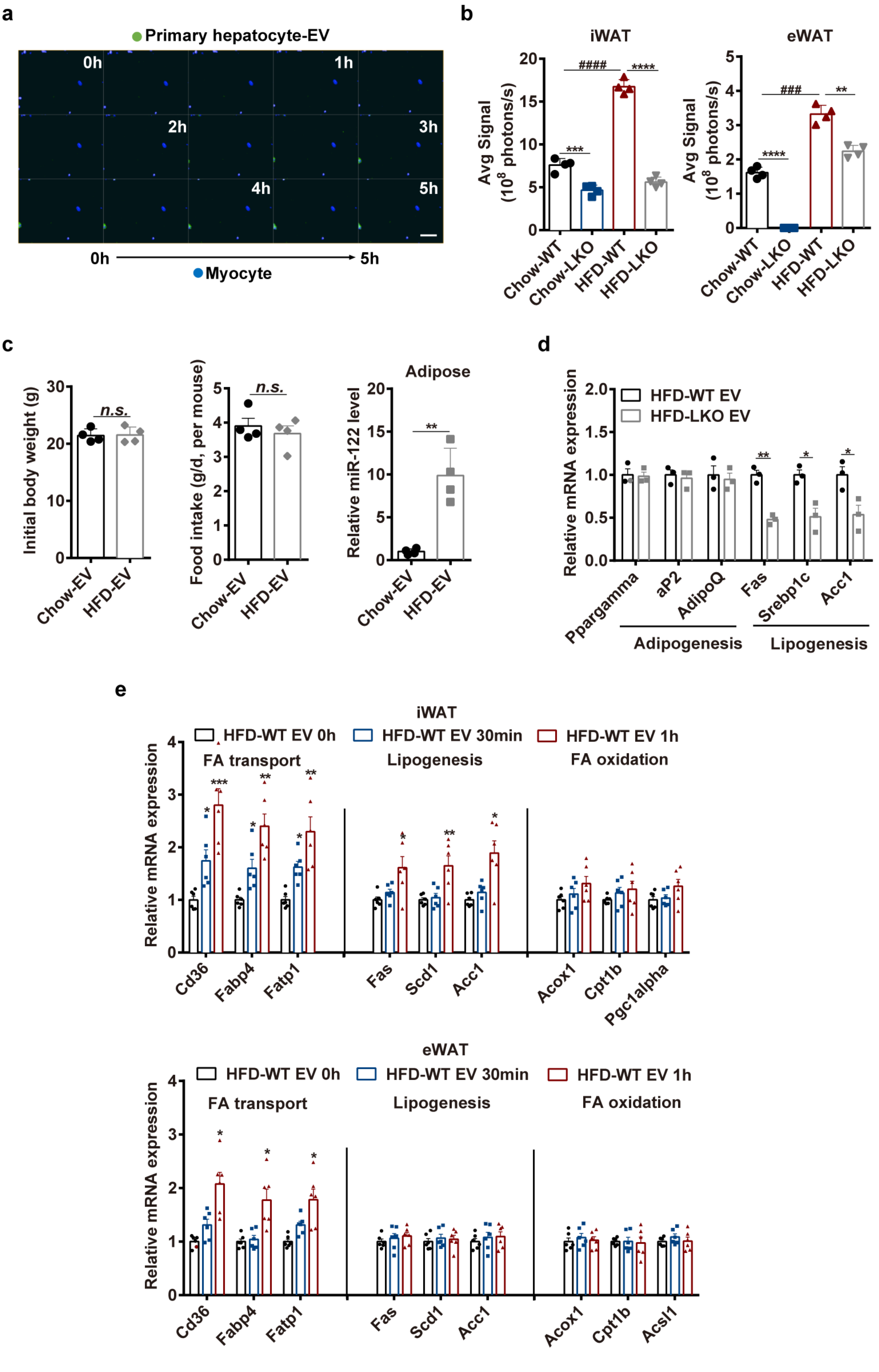
a
Primary hepatocyte-EV
0h
1h
2h
3h
4h
5h
0h
5h
Myocyte
b
iWAT
eWAT
Avg Signal (10^8 photons/s)
####
****
***
###
**
****
Chow-WT
Chow-LKO
HFD-WT
HFD-LKO
c
Initial body weight (g)
n.s.
Food intake (g/d, per mouse)
Adipose
Relative miR-122 level
**
Chow-EV
HFD-EV
d
HFD-WT EV
HFD-LKO EV
Relative mRNA expression
Ppargamma
aP2
AdipoQ
Fas
Srebp1c
Acc1
Adipogenesis
Lipogenesis
e
iWAT
HFD-WT EV 0h
HFD-WT EV 30min
HFD-WT EV 1h
FA transport
Lipogenesis
FA oxidation
Relative mRNA expression
Cd36
Fabp4
Fatp1
Fas
Scd1
Acc1
Acox1
Cpt1b
Pgc1alpha
eWAT
HFD-WT EV 0h
HFD-WT EV 30min
HFD-WT EV 1h
FA transport
Lipogenesis
FA oxidation
Relative mRNA expression
Cd36
Fabp4
Fatp1
Fas
Scd1
Acc1
Acox1
Cpt1b
Acsl1

Supplementary Figure 6. Hepatic EV uptake in myocytes and lipid metabolism genes in adipose tissue treated with hepatic EVs. Related to Fig. 5.

(**a**) Primary hepatocyte EV uptake in L6 myocytes after co-culture for 5 hours. (scale bar, 50 μm).

(**b**) Fluorescence intensity of the images in Fig. 5b was quantified using Living Image 3.1 software. (n=4 mice per group).

(**c**) The initial body weight, food intake amount and miR-122 levels in adipose from the mice from Fig. 5c. (n=4 mice per group).

(**d**) Expression of genes related to adipogenesis and lipogenesis in the 3T3-L1 preadipocytes from Fig. 5e. (n=3 biologically independent samples per group).

(**e**) Expression of genes related to fatty acid transport, lipogenesis and fatty acid oxidation in iWAT and eWAT of 8-week-old C57BL/6J mice following HFD-WT EV treatment for 0 hours, 0.5 hours, and 1 hour. (n=6 mice per group).

Data are presented as the mean ± SEM. Groups were analysed using an unpaired t test, *$P < 0.05$, **$P < 0.01$, ***$P < 0.001$; ###$P < 0.001$.

Source data are provided as a Source Data file.

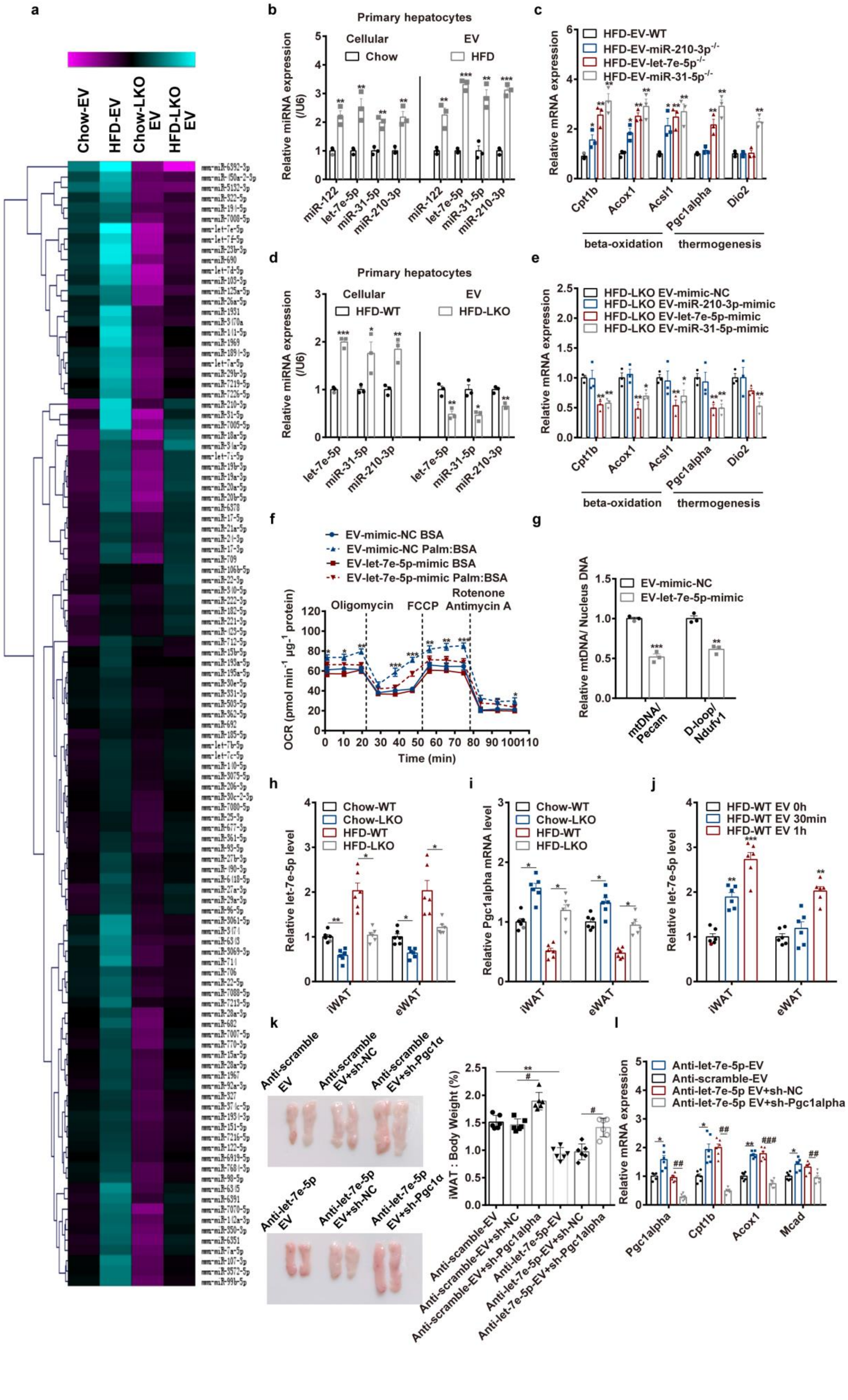

a
Chow-EV
HFD-EV
Chow-LKO EV
HFD-LKO EV
b
Primary hepatocytes
Cellular
EV
Chow
HFD
Relative miRNA expression (/U6)
miR-122
let-7e-5p
miR-31-5p
miR-210-3p
c
HFD-EV-WT
HFD-EV-miR-210-3p-/-
HFD-EV-let-7e-5p-/-
HFD-EV-miR-31-5p-/-
Relative mRNA expression
Cpt1b
Acox1
Acsl1
Pgc1alpha
Dio2
beta-oxidation
thermogenesis
d
Primary hepatocytes
Cellular
EV
HFD-WT
HFD-LKO
Relative miRNA expression (/U6)
e
HFD-LKO EV-mimic-NC
HFD-LKO EV-miR-210-3p-mimic
HFD-LKO EV-let-7e-5p-mimic
HFD-LKO EV-miR-31-5p-mimic
Relative mRNA expression
f
EV-mimic-NC BSA
EV-mimic-NC Palm:BSA
EV-let-7e-5p-mimic BSA
EV-let-7e-5p-mimic Palm:BSA
Oligomycin
FCCP
Rotenone Antimycin A
OCR (pmol min-1 μg-1 protein)
Time (min)
g
EV-mimic-NC
EV-let-7e-5p-mimic
Relative mtDNA/ Nucleus DNA
mtDNA/ Pecam
D-loop/ Ndufv1
h
Chow-WT
Chow-LKO
HFD-WT
HFD-LKO
Relative let-7e-5p level
iWAT
eWAT
i
Relative Pgc1alpha mRNA level
j
HFD-WT EV 0h
HFD-WT EV 30min
HFD-WT EV 1h
Relative let-7e-5p level
k
Anti-scramble EV
Anti-scramble EV+sh-NC
Anti-scramble EV+sh-Pgc1α
Anti-let-7e-5p EV
Anti-let-7e-5p EV+sh-NC
Anti-let-7e-5p EV+sh-Pgc1α
iWAT : Body Weight (%)
Anti-scamble-EV
Anti-scramble-EV+sh-NC
Anti-scramble-EV+sh-Pgc1alpha
Anti-let-7e-5p-EV
Anti-let-7e-5p-EV+sh-NC
Anti-let-7e-5p-EV+sh-Pgc1alpha
l
Anti-let-7e-5p-EV
Anti-scramble-EV
Anti-let-7e-5p EV+sh-NC
Anti-let-7e-5p EV+sh-Pgc1alpha
Relative mRNA expression
Pgc1alpha
Cpt1b
Acox1
Mcad

Supplementary Figure 7. The Profiles and Functions of MiRNAs from Hepatic EVs. (Related to Fig. 6.)

(**a**) Differential expression levels of EV miRNAs between mice fed a normal chow diet (Chow-EV) or an HFD (HFD-EV) and LKO mice fed a normal chow diet (Chow-LKO EV) or a HFD (HFD-LKO EV). Each group was loaded with 5 μg of EVs.

(**b**) Abundance of miR-122, let-7e-5p, miR-31-5p and miR-210-3p in primary hepatocytes and EVs secreted by mice fed a normal chow diet or an HFD. (n=3 biologically independent samples per group).

(**c**) Expression of genes related to fatty acid oxidation and thermogenesis in the 3T3-L1 preadipocytes from Fig. 6c. (n=3 biologically independent samples per group).

(**d**) Abundance of let-7e-5p, miR-210-3p and miR-31-5p in primary hepatocytes and EVs secreted by WT and LKO mice fed a HFD. (n=3 biologically independent samples per group).

(**e**) Expression of genes related to fatty acid oxidation and thermogenesis in the 3T3-L1 preadipocytes from Fig. 6e. (n=3 biologically independent samples per group).

(**f**) OCR of 3T3-L1 preadipocytes using fatty acids, as detected with the XF Palmitate-BSA FAO Substrate and the XF Cell Mito Stress Test. (n=5 biologically independent samples per group).

(**g**) Relative mtDNA of the 3T3-L1 preadipocytes from Fig. 6h. (n=3 biologically independent samples per group).

(**h-i**) Expression of let-7e-5p and Pgc1α in adipose tissue of mice (initial age 8 weeks) fed a normal chow diet or an HFD for 12 weeks. (n=6 mice per group).

(**j**) Expression of let-7e-5p in adipose tissue of 8-week-old C57BL/6J mice following treatment with EVs derived from hepatocytes from HFD-WT mice for 0 hours, 0.5 hours, and 1 hour. (n=6 mice per group).

(**k**) Comparison of iWAT fat pads in mice after treatment with anti-scramble-EV, anti-let-7e-5p-EVs, sh-NC or sh-Pgc1α. Percentage of the iWAT weight relative to the whole-body weight of the mice. (n=6 mice per group).

(**l**) Expression of genes related to lipid oxidation in iWAT of the mice from Supplementary Figure 7k. (n=6 mice per group).

Data are presented as the mean ± SEM. Groups were analysed using an unpaired t test, **P* < 0.05, ***P* < 0.01, ****P* < 0.001; #*P* < 0.05, ##*P* < 0.01, ###*P* < 0.001.

Source data are provided as a Source Data file.

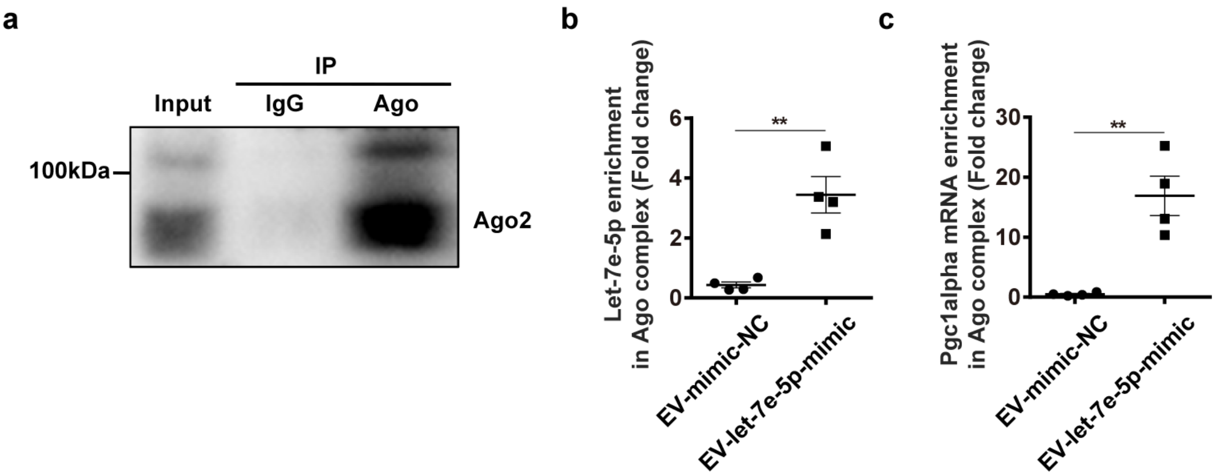

Supplementary Figure 8. Pgc1α from Adipocytes in Complex with Ago and Hepatic EV-let-7e-5p. (Related to Fig. 6.)

(**a**) Immunoprecipitation of Ago from lysed let-7e-5p$^{-/-}$ adipocytes treated with hepatocyte-derived EVs. Negative control mouse IgG was used as an IP control. (**b-c**) RNA ChIP analyses of Ago-miRNA complexes subjected to Ago pull-down. Total isolated RNA was analysed by qPCR for the abundance of let-7e-5p (**b**) and Pgc1α mRNA (**c**) bound with Ago protein in recipient cells that overexpressed let-7e-5p. Data are presented as the mean ± SEM. n = 4 biologically independent samples per group. $^{**}P < 0.01$, unpaired t test.

Source data are provided as a Source Data file.

Supplementary Table 1. Clinical Characteristics and Steatosis Severity Score of the Patients. Related to Fig. 7.

Steatosis score	Gender	BMI	Blood glucose (mmol/L)	Blood TG (mmol/L)	Number
3	Male	39-50	4.06-6.78	0.86-5.35	6
2	Male	31.2-47.1	4.3-7.93	1.51-5.57	9
1	Male	21.3-38.3	4.52-7.6	1.27-5.83	13

Supplementary Table 2. Sequence of SiRNAs and Primers.

**Gene**	**Sequence (5’-3’)**
Scramble-sense	UUCUCCGAACGUCACGUdTdT
Scramble-antisense	ACGUGACACGUUCGGAGAAdTdT
si*Ggpps*920-sense	CGCCAGAGAACAGAGAAUATT
si*Ggpps*920-antisense	UAUUCUCUGUUCUCUGGCGTT
sh-*Pgc1α*	GGTGGATTGAAGTGGTGTAGA
18S-F	GTCTGTGATGCCCTTAGATG
18S-R	AGCTTATGACCCGCACTTAC
CD36-F	ATGGGCTGTGATCGGAACTG
CD36-R	GTCTTCCCAATAAGCATGTCTCC
Ldlr-F	TCCCTGGGAACAACTTCACC
Ldlr-R	CACTCTTGTCGAAGCAGTCAG
Lrp-F	CACAACCTCAACGTCATCCTG
Lrp-R	AGCACATTGTACTCCTGGATCTT
Fas-F	AGGTGGTGATAGCCGGTATGT
Fas-R	TGGGTAATCCATAGAGCCCAG
Scd1-F	TTCTTGCGATACACTCTGGTGC
Scd1-R	CGGGATTGAATGTTCTTGTCGT
Acc1-F	GATGAACCATCTCCGTTGGC

Acc1-R	CCCAATTATGAATCGGGAGTGC
Acox1-F	GCCTGCTGTGTGGGTATGTCATT
Acox1-R	GTCATGGGCGGGTGCAT
Cpt1a-F	CTCAGTGGGAGCGACTCTTCA
Cpt1a-R	GGCCTCTGTGGTACACGACAA
Cpt1b-F	GCACACCAGGCAGTAGCTTT
Cpt1b-R	CAGGAGTTGATTCCAGACAGGTA
Cpt2-F	TTCACCACTAAGAACGGTTCG
Cpt2-R	CTCCAAGGATGACACTGATTTCA
Acadl-F	TCTTTTCCTCGGAGCATGACA
Acadl-R	GACCTCTCTACTCACTTCTCCAG
Fabp4-F	AAGGTGAAGAGCATCATAACCCT
Fabp4-R	TCACGCCTTTCATAACACATTCC
Fabp1-F	ATGAACTTCTCCGGCAAGTACC
Fabp1-R	CTGACACCCCCTTGATGTCC
Fabp3-F	ACCTGGAAGCTAGTGGACAG
Fabp3-R	TGATGGTAGTAGGCTTGGTCAT
Acsl1-F	TGCCAGAGCTGATTGACATTC
Acsl1-R	GGCATACCAGAAGGTGGTGAG
IL8-F	CAAGGCTGGTCCATGCTCC
IL8-R	TGCTATCACTTCCTTTCTGTTGC

Hgf-F	ATGTGGGGGACCAAACTTCTG
Hgf-R	GGATGGCGACATGAAGCAG
Tgf-β-F	CTCCCGTGGCTTCTAGTGC
Tgf-β-R	GCCTTAGTTTGGACAGGATCTG
Fgf21-F	CTGCTGGGGGTCTACCAAG
Fgf21-R	CTGCGCCTACCACTGTTCC
Cre-F	TGCCACGACCAAGTGACAGCAATG
Cre-R	AGAGACGGAAATCCATCGCTCG
Loxp-F	AATTGTGTGTGGTAGGGGTA
Loxp-R	AACTTGCTTCAGAACTGAGC
Srebp1c-F	GATGTGCGAACTGGACACAG
Srebp1c-R	GCATGTCTTCGATGTCGTTCAAA
Lpl-F	GGGAGTTTGGCTCCAGAGTTT
Lpl-R	TGTGTCTTCAGGGGTCCTTAG
Ppar γ-F	TCGCTGATGCACTGCCTATG
Ppar γ-R	GAGAGGTCCACAGAGCTGATT
AdipoQ-F	TGTTCCTCTTAATCCTGCCCA
AdipoQ-R	CCAACCTGCACAAGTTCCCTT
Pgc1α-F	CCCTGCCATTGTTAAGACC
Pgc1α-R	TGCTGCTGTTCCTGTTTTC
Dio2-F	AATTATGCCTCGGAGAAGACCG

Dio2-R	GGCAGTTGCCTAGTGAAAGGT
Ggpps-F human	CCAGGTAAACAAGTGAGAACCAA
Ggpps-R human	CGTCGGAGTTTTGAGTTGTCT
Ggpps-F mouse	TTTTGCATACACTCGACACACT
Ggpps-R mouse	ACCACAGGCCTCAATTTGTTTGT
D-loop non-coding region-F	GGTTCTTACTTCAGGGCCATCA
D-loop non-coding-R	GATTAGACCCGTTACCATCGAGAT
Ndufv1-F	CTTCCCCACTGGCCTCAAG
Ndufv1-R	CCAAAACCCAGTGATCCAGC
mtDNA-F	CCTATCACCCTTGCCATCAT
mtDNA-R	GAGGCTGTTGCTTGTGTGAC
Pecam-F	ATGGAAAGCCTGCCATCATG
Pecam-R	TCCTTGTTGTTCAGCATCAC
