# Supplementary material for: Liver governs adipose remodelling via extracellular vesicles in response to lipid overload
Source: Nat Commun. 2020 Feb 5;11:719. doi: 10.1038/s41467-020-14450-6 (PMC7002740; doi:10.1038/s41467-020-14450-6)
Supplement: Supplementary file 3 — Description of Additional Supplementary Files [file 41467_2020_14450_MOESM3_ESM.pdf]

Description of Additional Supplementary Files

File name: Supplementary Movie 1

Description: Time Lapse of Hepatic EVs Entering Adipocytes.
