# Supplementary material for: Liver governs adipose remodelling via extracellular vesicles in response to lipid overload
Source: Nat Commun. 2020 Feb 5;11:719. doi: 10.1038/s41467-020-14450-6 (PMC7002740; doi:10.1038/s41467-020-14450-6)
Supplement: Supplementary file 5 — Reporting Summary [file 41467_2020_14450_MOESM5_ESM.pdf]

nature research

Corresponding author(s): Chaojun Li

Last updated by author(s): Aug 30, 2019

Reporting Summary

Nature Research wishes to improve the reproducibility of the work that we publish. This form provides structure for consistency and transparency in reporting. For further information on Nature Research policies, see Authors & Referees and the Editorial Policy Checklist .

Statistics

For all statistical analyses, confirm that the following items are present in the figure legend, table legend, main text, or Methods section.

n/a	Confirmed	
☐	☒	The exact sample size (n) for each experimental group/condition, given as a discrete number and unit of measurement
☐	☒	A statement on whether measurements were taken from distinct samples or whether the same sample was measured repeatedly
☐	☒	The statistical test(s) used AND whether they are one- or two-sided<br>*Only common tests should be described solely by name; describe more complex techniques in the Methods section.*
☐	☒	A description of all covariates tested
☐	☒	A description of any assumptions or corrections, such as tests of normality and adjustment for multiple comparisons
☐	☒	A full description of the statistical parameters including central tendency (e.g. means) or other basic estimates (e.g. regression coefficient) AND variation (e.g. standard deviation) or associated estimates of uncertainty (e.g. confidence intervals)
☐	☒	For null hypothesis testing, the test statistic (e.g. F, t, r) with confidence intervals, effect sizes, degrees of freedom and P value noted<br>*Give P values as exact values whenever suitable.*
☒	☐	For Bayesian analysis, information on the choice of priors and Markov chain Monte Carlo settings
☒	☐	For hierarchical and complex designs, identification of the appropriate level for tests and full reporting of outcomes
☒	☐	Estimates of effect sizes (e.g. Cohen's d, Pearson's r), indicating how they were calculated

Our web collection on statistics for biologists contains articles on many of the points above.

Software and code

Policy information about availability of computer code

Data collection	Immunofluorescence microscopy images were acquired using Leica TCS SP8 microscope and High Content Analysis Operetta. Western blot data was acquired digitally by Image LAB (Bio-Rad) software. qPCR data was acquired using the Applied Biosystems 7500 Fast Real Time PCR system software (Thermo).
Data analysis	Data were analyzed using Prism Graphpad version 6 and 7. Microscopy images analysis was performed using ImageJ software. Densitometric analysis of western blots was performed with ImageJ software.

For manuscripts utilizing custom algorithms or software that are central to the research but not yet described in published literature, software must be made available to editors/reviewers. We strongly encourage code deposition in a community repository (e.g. GitHub). See the Nature Research guidelines for submitting code & software for further information.

Data

Policy information about availability of data

All manuscripts must include a data availability statement. This statement should provide the following information, where applicable:

- Accession codes, unique identifiers, or web links for publicly available datasets
- A list of figures that have associated raw data
- A description of any restrictions on data availability

All experimental data from mice models and scanned images of western blots are provided in the source data file. All data sets generated and/or analyzed in the current study are available from the corresponding author upon reasonable request.

Field-specific reporting

Please select the one below that is the best fit for your research. If you are not sure, read the appropriate sections before making your selection.

☒ Life sciences ☐ Behavioural & social sciences ☐ Ecological, evolutionary & environmental sciences

For a reference copy of the document with all sections, see nature.com/documents/nr-reporting-summary-flat.pdf

Life sciences study design

All studies must disclose on these points even when the disclosure is negative.

Sample size	No statistical methods were used to predetermine sample size (n). Number of sample was determined based on experimental approach, availability, feasibility required to obtain definitive results.
Data exclusions	Exclusion criteria for animals were applied in case of death, cannibalism and the presence of severe clinical alteration of vita physiological functions. Exclusion criteria for samples were applied in case of histological artifacts (freeze- and cut-damaged tissues), RNA and protein degradation. Exclusion criteria for clinical data were applied in case of females and patients with evidence for viral hepatitis, hemochromatosis, or alcohol consumption (>20 g/d for females and>30 g/d for males).
Replication	All of experiments have been successfully repeated at least three times and/or with sufficient cells/animals per group to demonstrate statistical significance. All experiments were statistically analyzed.
Randomization	The mice were randomly assigned to each experimental/control group.
Blinding	Each mouse was assigned a code number to enable blinded chow diet or high fat diet; when applicable, experimenters were blinded to the nature of the samples by using number codes until final data analysis was performed.

Reporting for specific materials, systems and methods

We require information from authors about some types of materials, experimental systems and methods used in many studies. Here, indicate whether each material, system or method listed is relevant to your study. If you are not sure if a list item applies to your research, read the appropriate section before selecting a response.

Materials & experimental systems

n/a	Involved in the study	
☐	☒	Antibodies
☐	☒	Eukaryotic cell lines
☒	☐	Palaeontology
☐	☒	Animals and other organisms
☒	☐	Human research participants
☐	☒	Clinical data

Methods

n/a	Involved in the study	
☒	☐	ChIP-seq
☐	☒	Flow cytometry
☒	☐	MRI-based neuroimaging

Antibodies

Antibodies used	CD63 (Abcam, ab68418), TSG101 (Abcam, ab125011), syntenin-1 (Abcam, ab205861), Rab27a (Abcam, ab55667), Rab5 (Abcam, ab18211), CD81 (Santa Cruz Biotechnology, sc-166029) , Pgc1a (Santa Cruz Biotechnology, sc-518038), GGPPS (Santa Cruz Biotechnology, sc-271680), b-actin (Santa Cruz Biotechnology, sc-47778), CALR (Cell Signaling Technology, 12238), H3 (Cell Signaling Technology, 4499), Calnexin (Cell Signaling Technology, 2433) and AGO2 (Proteintech, 10686-1-AP).
Validation	All of antibodies were validated by vendors or other researchers. We based specificity on their provided data sheets.

Eukaryotic cell lines

Policy information about cell lines

Cell line source(s)	3T3-L1 cell lines (gift from Qi-Qun Tang, Fudan Univeristy) are originated from Lane MD (Department of Biological Chemistry, Johns Hopkins University School of Medicine).
Authentication	The cell line used was extracted DNA for comparison in DNA database for authentication by Qi-Qun Tang.
Mycoplasma contamination	3T3-L1 cell line was maintained under the recommended culture conditions and media requirements. Mycoplasma detection was performed in accordance with department protocols (and tested negative).

Commonly misidentified lines (See ICLAC register)	None

Animals and other organisms

Policy information about studies involving animals; ARRIVE guidelines recommended for reporting animal research

Laboratory animals	Stated in Materials and Methods under "Animals": male C57BL/6J mice purchased from the Model Animal Research Center of Nanjing University for our study. Liver-specific Ggpps knockout mice were generated by crossing Ggpps-floxed mice with Mx1-Cre transgenic mice. For the HFD model, WT and L-Ggpps KO mice were fed the HFD at 8 weeks of age for 12 weeks.
Wild animals	The study did not involved in wild animals.
Field-collected samples	The study did not involved in samples collected from the field.
Ethics oversight	All animal procedures were carried out in accordance with the approval of the Animal Care and Use Committee at the Model Animal Research Center of Nanjing University in Nanjing, China, using approved protocols from the institutional animal care committee (#CS20).

Note that full information on the approval of the study protocol must also be provided in the manuscript.

Clinical data

Policy information about clinical studies

All manuscripts should comply with the ICMJEguidelines for publication of clinical research and a completedCONSORT checklist must be included with all submissions.

Clinical trial registration	This study was registered in International Clinical Trial Registry Platform (ICTRP), with the clinical trial number NCT03296605.
Study protocol	Plasma samples were obtained before surgery and liver tissue samples were obtained during surgery. Some liver tissue samples were analyzed immediately, and some samples were put into liquid nitrogen and stored at -80 °C. Specimen was stored in the Nanjing Multicenter Biobank, the Biobank of Nanjing Drum Tower Hospital, the Affiliated Hospital of Nanjing University Medical School.
Data collection	The participants were recruited from April 2017 to February 2018. We conducted this study from February 2018.
Outcomes	The analysis of Ggpps expression level and TG content of liver samples showed that Ggpps expression levels directly correlated with TG content in the livers of patients, suggesting that Ggpps in liver may response to lipid overload in accordance with the data of mice. Some miRNAs were also enriched in the plasma of NAFLD patients with BMI > 30 compared with their expression in NAFLD patients with BMI < 30, suggesting that hepatocyte-derived EV miRNAs maybe responsible for liver-driven adipose remodeling in human.

Flow Cytometry

Plots

Confirm that:

- [x] The axis labels state the marker and fluorochrome used (e.g. CD4-FITC).
- [x] The axis scales are clearly visible. Include numbers along axes only for bottom left plot of group (a 'group' is an analysis of identical markers).
- [x] All plots are contour plots with outliers or pseudocolor plots.
- [x] A numerical value for number of cells or percentage (with statistics) is provided.

Methodology

Sample preparation	Cells were collected by trypsinization and pelleted by centrifugation at 1000 x g for 3 min. The cells were stained with annexin V (Beyotime, Nantong, China; 1:1000 dilution) and propidium iodide (Beyotime, Nantong, China; 1:1000 dilution) in annexin V binding buffer containing 10 mM HEPES (pH 7.4), 150 mM NaCl, 5 mM KCl, 1 mM MgCl2, and 1.8 mM CaCl2 for 15 min on ice and then subjected to flow cytometric analysis.
Instrument	FACSCALLBUR flow cytometer (BD Biosciences)
Software	FlowJo
Cell population abundance	N/A
Gating strategy	Gating was performed using FITC and PE.

- [x] Tick this box to confirm that a figure exemplifying the gating strategy is provided in the Supplementary Information.
